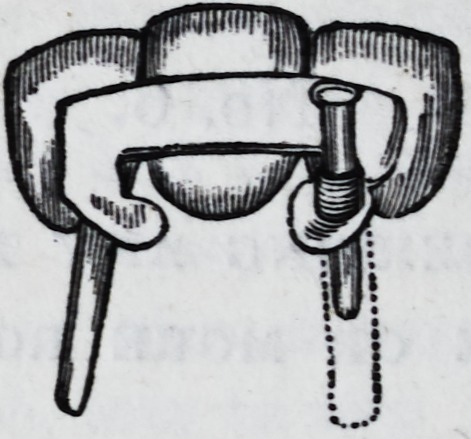# Contributions to Operative and Mechanical Dentistry

**Published:** 1845-03

**Authors:** W. H. Elliot

**Affiliations:** Fellow of the American Society of Dental Surgeons.


THE AMERICAN

### JOURNAL OF DENTAL SCIENCE.

Vol. V.] MARCH, 1844. [No. 3.

### ARTICLE I.

*Contributions to Operative and Mechanical Dentistry.* By W. H. Elliot, D. D. S., Fellow of the American Society of Dental Surgeons.

### No. 6.

### A NEW METHOD OF INSERTING ANY NUMBER OF TEETH UPON ONE OR MORE ROOTS.

The injurious effects of placing plates immovably upon the gum, so that they cannot be taken off for the purpose of cleansing them, should be a sufficient apology for making it a principle never to attach plates of any description either to the roots or crowns of the natural teeth, in such a way that they may not be moved at pleasure by the patient.

It is not necessary to state here, how or why, the gums under and around such teeth are kept in a state of irritable inflammation, or its consequent effects upon the delicate membranes surrounding the roots of the adjacent teeth. These are already understood, and few, it is hoped, will hesitate to embrace any method that promises to do away with so many evil consequences.

No advantage, whatever, can be gained by covering a portion of the gum with a plate, which is permanently attached to the roots of teeth for its support; for the gum will always yield sufficiently to pressure to leave the whole force to be sustained by the roots; and when such plates are used, they merely serve as connecting links between the different parts of the work.

The cut below represents the writer's "method of inserting any number of teeth upon one or more roots," and also his method of proceeding when the roots are so convergent or divergent, that the pivots cannot be made to enter the openings at the same time. By reference to the cut, it may be seen that one of the pivots is detached from the rest of the work, and by being made to fit snugly through a short tube, it counteracts any lateral force equally as well as the pivot, which is permanently soldered to the plate. In case the roots stand exactly parallel, a detached pivot is not required, but if the roots be not parallel, one of the pivots must be detached; and when the work is placed in the mouth, the permanent pivot must first be secured to its root, and then the detached pivot may be forced through the tube into the root to which it belongs.

In preparing the roots, it is necessary to file them a little below the edge of the gum as in case of a single pivot-tooth. The opening in the roots should be drilled just large enough to receive the golden-pivots which are to be used for the support of the work; if a detached pivot be used, the hole in one root must be sufficiently large to receive the tube through which the pivot is to slide.

If the roots be not exactly parallel, in withdrawing the wax from the mouth, one or both of the iron pivots* will be found slightly moved, so that a small opening will appear between the pivot and the wax upon one side, the disturbed pivots should be carefully moved into their true positions, and when this is done, the opening in the wax will all appear upon the other side of the pivot. On removing the wax from the model, the iron pivots will be found to indicate the exact position and direction of the

* For the writer's method of taking casts in such cases, the reader is referred to the Journal, vol. 4, page 165.

openings in the roots; an end which cannot be obtained by other means.

Models for this purpose should be composed of one part of *talc*, and two parts of gypsum; talc is preferable to sand, because being finer, it makes a more perfect model, and it is necessary that it be unchangeable by heat.

After the model has become sufficiently hard, the iron-pivots may be carefully withdrawn from it, and their places supplied by the golden pivots or pivot and tube, as the case may require. The tube should be long enough to rise above the plate at least one line, if practicable, as all the tube below the plate is to be cut off, after the work is soldered.

Two very thin platina or gold plates may now be procured, about three-fourths of an inch in length, and wide enough to cover the end of the root, after a hole has been made through the end of each plate, they may be slipped over the pivots, or pivot and tube, and brought to a perfect fit to the model by being pressed down upon it. This may be done without injuring the model, if the plates be thin and well annealed. The free end of these plates which should project backward towards the palate may now be bent down near the model, and fastened by laying on more of the mixture of talc and plaster of paris. And then, when the teeth have been selected, fitted, and fastened to the model by cement,* more of the mixture may be put upon the model so as to cover the anterior surfaces of the teeth.

Those teeth nearest the pivots may now have perpendicular backs placed upon them, reaching down to the small plates upon the ends of the roots; while the other teeth should have horizontal backs, stretching across from one perpendicular back to the other, so as to connect the different parts of the work together. The cutting edges of the teeth may now be covered with the mixture, and the whole bound lightly together by a few turns of fine binding wire.

When soldered, the plates may be cut off and trimmed to the exact size of the ends of the roots, and all that portion of the tube, represented by the dotted lines in the cut, may also be cut off.

Work of this kind must always depend upon the solder for strength, and, on that account, thin plates, which are much more easily wrought, are preferable.

* See Journal, vol. 5, page 87.

## Figures and Tables

**Figure f1:**